# Studies on allergic diseases and B cells in the past 20 years: a bibliometric analysis via CiteSpace and VOSviewer

**DOI:** 10.3389/fimmu.2026.1760388

**Published:** 2026-05-14

**Authors:** Weiyuan Mai, Xiaoqu Chen, Wanlin Ye, Guangshen Zhang, Wenyu Liang

**Affiliations:** 1 Department of Otolaryngology, Maoming Maternal and Child Health Hospital, Maoming, Guangdong, China; 2 Department of Surgery, Maoming Maternal and Child Health Hospital, Maoming, Guangdong, China

**Keywords:** allergen immunotherapy, allergic diseases, B cells, bibliometric analysis, Citespace, VOSviewer

## Abstract

**Background:**

Allergic diseases represent a growing global health burden, and B cells have emerged as central yet incompletely defined regulators of IgE-mediated immunity and tolerance. There is currently a lack of bibliometric research on allergic diseases and B cells.

**Methods:**

Records were retrieved from the Science Citation Index Expanded of the Web of Science Core Collection (WoSCC) and PubMed. After applying language, time, and article-type restrictions, 3, 084 WoSCC articles and 71 PubMed-indexed clinical trials were included. CiteSpace (v6.4.R1), VOSviewer (v1.6.20), and Excel were used to analyze publication and citation trends, journals, countries, institutions, authors, keyword co-occurrence, and burst terms, and to visualize co-authorship and thematic networks.

**Results:**

Annual publications and citations increased steadily, indicating sustained academic interest. Output was concentrated in leading allergy and immunology journals and in institutions from Europe and the United States, although contributions from other regions have increased over time. Keyword clustering identified stable cores around ‘food allergy, ‘ ‘asthma, ‘ and ‘plasma cells, ‘ while burst and overlay analyses highlighted ‘regulatory B cells’ and ‘innate lymphoid cells’ as emerging research hotspots. These bibliometric patterns indicate growing attention to tissue-associated B-cell biology, immune regulation, and allergen immunotherapy. PubMed clinical-trial records further suggest increasing translational interest in B-cell-related interventions for allergic diseases.

**Conclusions:**

Over the past 20 years, research on allergic diseases and B cells has evolved from descriptive immunoglobulin E (IgE) associations toward a tissue- and systems-level exploration of B-cell biology. This bibliometric analysis delineates research hotspots and translational themes, Future progress will require large, multicenter studies with standardized B-cell phenotyping and functional endpoints to support precision medicine in allergy.

## 1 Introduction

Allergic diseases, encompassing asthma, allergic rhinitis, atopic dermatitis, food allergy, drug hypersensitivity, and chronic urticaria, have emerged as a major global public health concern (1). The prevalence of these conditions has risen steadily, particularly in children and young adults, and is accompanied by increasing disease complexity, severity, and socio-economic burden (2). Allergic rhinitis alone affects hundreds of millions of individuals worldwide and remains a leading cause of morbidity and healthcare utilization (3), whereas food allergy now affects up to 10% of children in high-income countries and imposes substantial psychosocial and economic costs on patients and their families (4). Despite advances in biologic therapies targeting type 2 cytokines, allergen immunotherapy, and improved environmental control (5), no definitive cure exists for most allergic diseases, and many patients continue to experience persistent symptoms or only partial responses to treatment (6). Against this backdrop, research on allergic diseases has expanded over the past two decades to dissect the cellular and molecular mechanisms that drive IgE-mediated hypersensitivity responses, with B cells emerging as key, yet incompletely understood, regulators of these processes.

In allergic diseases, B cells have historically been regarded primarily as sources of allergen-specific IgE, the antibody class that arms mast cells and basophils for immediate hypersensitivity reactions (7). Allergen exposure within a type 2-polarized cytokine milieu—dominated by interleukin IL-4 and IL-13—drives class-switch recombination to IgE, giving rise to IgE-producing plasma cells (8). These antibodies bind with high affinity to FcϵRI on mast cells and basophils, such that subsequent allergen encounter triggers degranulation and the release of histamine, lipid mediators, and cytokines that underlie the acute symptoms of asthma, allergic rhinitis, urticaria, and anaphylaxis (9). However, B cells are now recognized as active regulators of allergic inflammation beyond IgE secretion, acting as antigen-presenting cells, shaping T-cell differentiation, producing pro-inflammatory cytokines, and modulating immune tolerance (10). More broadly, B lymphocytes are a core component of the adaptive immune system, responsible for antigen presentation, cytokine production, and the production of antibody-secreting plasma cells and long-lived memory B cells (11). Recent reviews emphasize that B-cell development, from hematopoietic stem cells through transitional and immature stages to germinal center and memory compartments, is tightly controlled by coordinated transcriptional programs and microenvironmental signals (12).

Among B-cell subsets, regulatory B cells (Bregs) have received increasing attention in studies of allergic diseases. Prior experimental and clinical reports suggest that altered Breg frequency or function may be associated with asthma, atopic dermatitis, allergic rhinitis, and food allergy (13), although their phenotype, stability, and disease-specific roles remain incompletely defined (14). In the context of allergen immunotherapy (AIT), the literature has also described increases in allergen-specific immunoglobulin G4 (IgG4), Bregs, and regulatory T cells, indicating that immune regulation is an important theme in this field (15).

Recent work has further complicated the traditional view of IL-10 as a purely anti-inflammatory cytokine. In a house dust mite (HDM)–induced asthma model, B-cell-derived IL-10, modulated by the transcription factor B-cell lymphoma 3 (Bcl-3), was shown to promote allergic sensitization by enhancing epithelial and dendritic cell responses to allergen (16); Bcl-3-deficient mice exhibited elevated IL-10 and exaggerated HDM-induced asthma (17). These observations imply that B-cell cytokine networks are context-dependent, with IL-10 exerting either regulatory or pro-allergic functions depending on the stage and tissue site of the response. Disentangling these roles and defining precisely which B-cell subsets exert protective versus pathogenic effects in human allergic disease remain major challenges.

Asthma exemplifies the complexity of B-cell involvement in the pathogenesis of allergic disease. Although asthma has traditionally been considered a T helper 2 (Th2)–driven airway disorder, severe and non-atopic asthma phenotypes reveal substantial heterogeneity in cellular contributors and endotypes (18). Studies integrating human samples and animal models show that B cells participate in airway inflammation by producing IgE and other antibody isotypes, presenting allergen to T cells, and modulating the activity of innate lymphoid cells, dendritic cells, and epithelial cells (19). Regulatory B cells and IgE^+^ B cells have both been detected in the airways and peripheral blood of patients with allergic asthma, but their abundance and functional properties vary with disease severity and treatment (20). Beyond asthma, B cells have been implicated in the pathogenesis of chronic spontaneous urticaria, atopic dermatitis, and IgE-mediated drug and food allergies (21). In chronic spontaneous urticaria and some forms of atopic dermatitis, small observational studies have suggested benefit from B-cell–depleting therapies such as rituximab, highlighting an autoantibody-driven component and further underscoring the diversity of B-cell effector functions in allergy (22).

Given their central roles in allergen sensitization, effector responses, and immune tolerance, B cells are increasingly considered attractive therapeutic targets in allergic diseases (23). In addition to conventional allergen immunotherapy, which induces shifts from IgE to IgG4 and expansion of regulatory T and B cells, several emerging strategies explicitly focus on modulating B-cell compartments (24). These include B-cell–depleting antibodies (25), agents that interfere with B-cell receptor (BCR) signaling (26), therapies targeting cytokine pathways critical for class switching (27), and approaches that seek to selectively delete or re-educate pathogenic memory B cells while sparing protective immunity (28). Despite increasing research, no comprehensive bibliometric analysis has systematically mapped the intellectual structure, evolution, and clinical translation of research on allergic diseases and B cells over the past two decades. Therefore, by systematically describing the knowledge structure and temporal evolution of allergic diseases and B cell research over the past two decades, we aim to provide a macro complement to mechanism and clinical research in this field.

## 2 Materials and methods

### 2.1 Data sources and search strategy

Records were retrieved from the Science Citation Index Expanded (SCI-E) of the Web of Science Core Collection (WoSCC) on 2025-10–01 to minimize database-update bias (29). The search covered the period from 2005-01–01 to 2024-12–31 and initially identified 4, 990 WoSCC records. After application of the predefined eligibility criteria, 3, 084 English-language WoSCC records were retained for bibliometric analysis. The complete executed WoSCC search string, field tags, filters, and controlled vocabulary or keyword combinations are provided in **Supplementary Table 1**.

To complement the bibliometric dataset with clinically oriented literature, PubMed was searched for clinical-trial records over the same time period (30), yielding 71 eligible records. The full PubMed search strategy, including Medical Subject Headings (MeSH) terms and filters, is provided in **Supplementary Table 2**. Three reviewers (WM, XC, and YW) independently screened titles, abstracts, and full texts. Eligible WoSCC document types were articles and review articles whose primary focus concerned allergic diseases and B cells. Excluded records comprised editorials, letters, case reports, conference abstracts, proceedings papers, notes, book chapters, guidelines, corrections, retractions, and unrelated publications. Disagreements were resolved by consensus with a fourth reviewer (GZ). Inter-rater reliability during screening was assessed using Fleiss’kappa (K >0.9, **Supplementary Table 4**). Four reviewers (WM, XC, YW, and GZ) extracted journal title, impact factor, publication year, countries, affiliations, author keywords, cited references, and citation counts, and discrepancies were adjudicated by the senior author (WL).

### 2.2 Data analysis

We conducted bibliometric analyses using advanced scientific-knowledge mapping and visualization tools: CiteSpace (v6.4.R1), VOSviewer (v1.6.20), and Microsoft Excel. In CiteSpace, we generated the dual-map overlay of journals, temporal evolution of keyword clusters, and keyword burst detection. In VOSviewer, we constructed and visualized country and institution-level co-authorship networks and keyword co-occurrence maps.

## 3 Results

### 3.1 Publication and citation landscape

**Figure 1:**
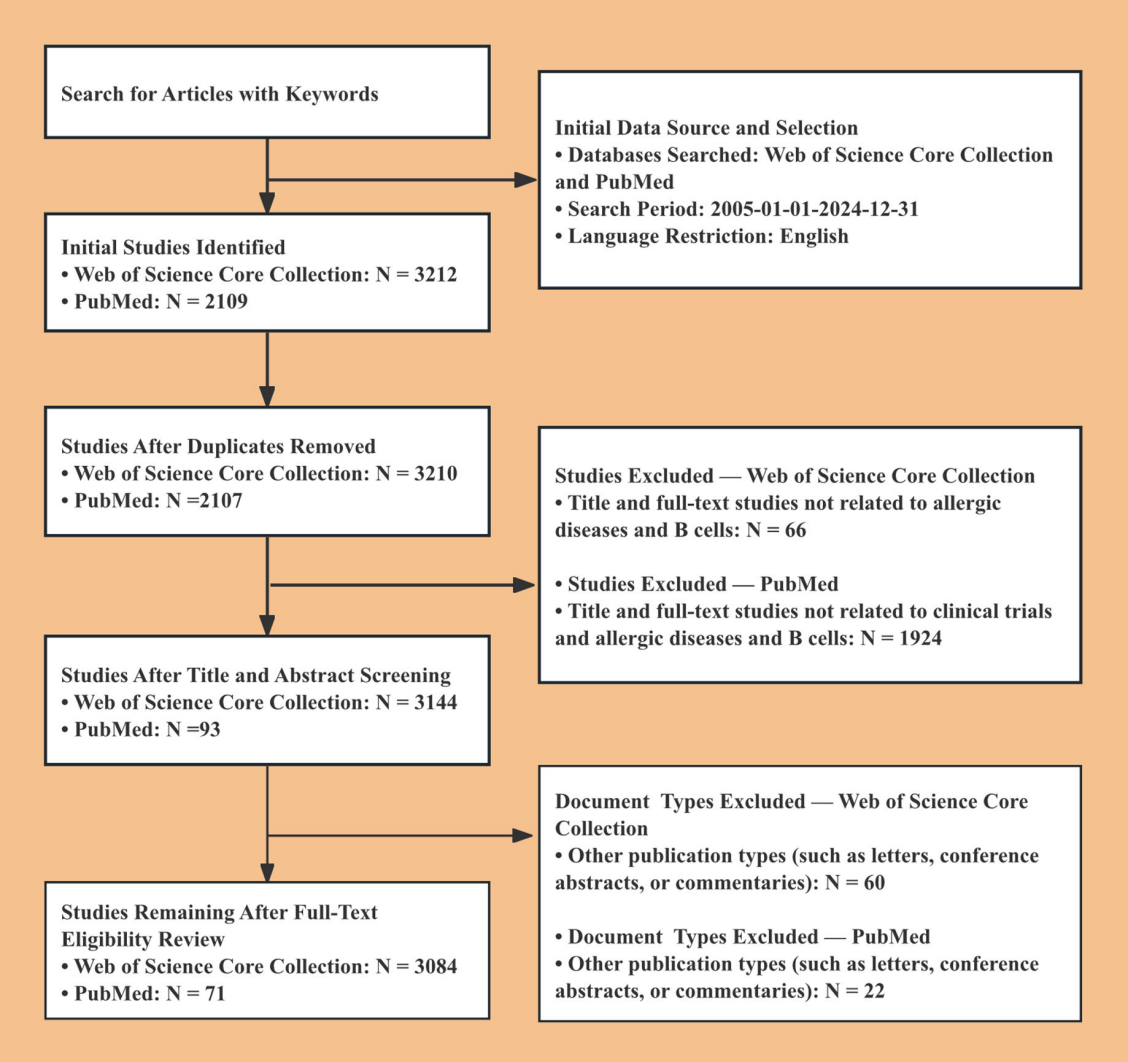
Flowchart outlining study identification, eligibility screening, and final inclusion of publications from WoSCC and PubMed.

A total of 4, 990 WoSCC records were initially retrieved for the study period. After applying the predefined time window, language, and document-type eligibility criteria, 3, 084 records were included in the bibliometric analysis, comprising 2, 319 articles and 765 review articles. **Figure 1** presents the study-selection flowchart and should explicitly distinguish the initial retrieval count from the final analytic dataset.

**Figure 2:**
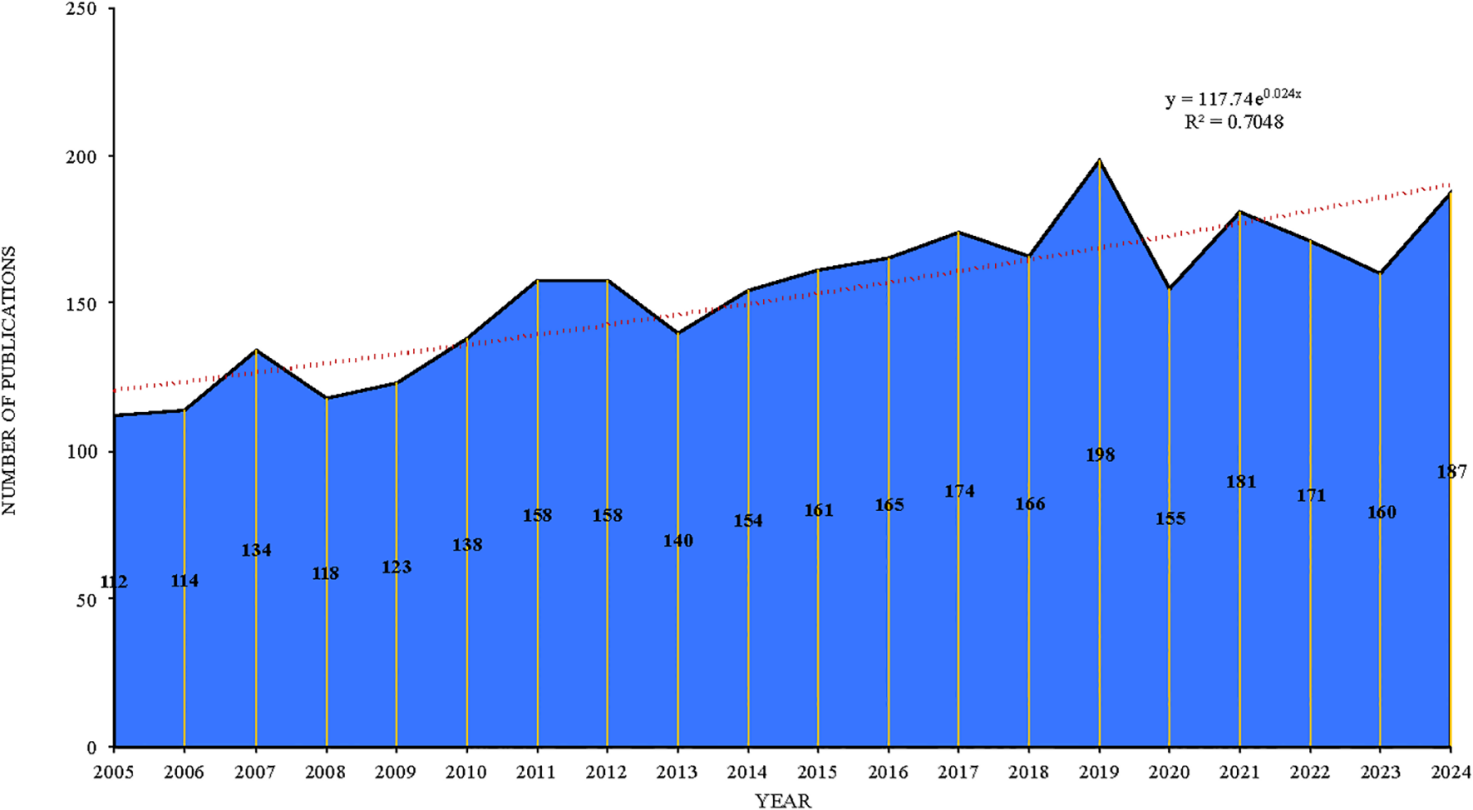
Number of annual publications and citation frequency relating to allergic diseases and B cells from 2005 to 2024.

We plotted the number of publications and citations by year to analyze trends in allergic disease and B-cell research over the past 20 years. As **Figure 2** shows, the number of published articles in this field has continued to increase over the past 20 years. In particular, the average number of articles published in this field reached 112 after 2005, exceeded 150 after 2011, and peaked in 2019 with 198 articles published. The citation frequency of articles has also increased, with more than 9000 citations in 2019. According to the Web of Science citation report, after excluding self-citations the corpus accrued 99, 308 citations, with a total of 133, 505 citations overall; the average citations per publication were 46.11, and the h-index was 160.

### 3.2 Productive journals and influential articles

**Figure 3:**
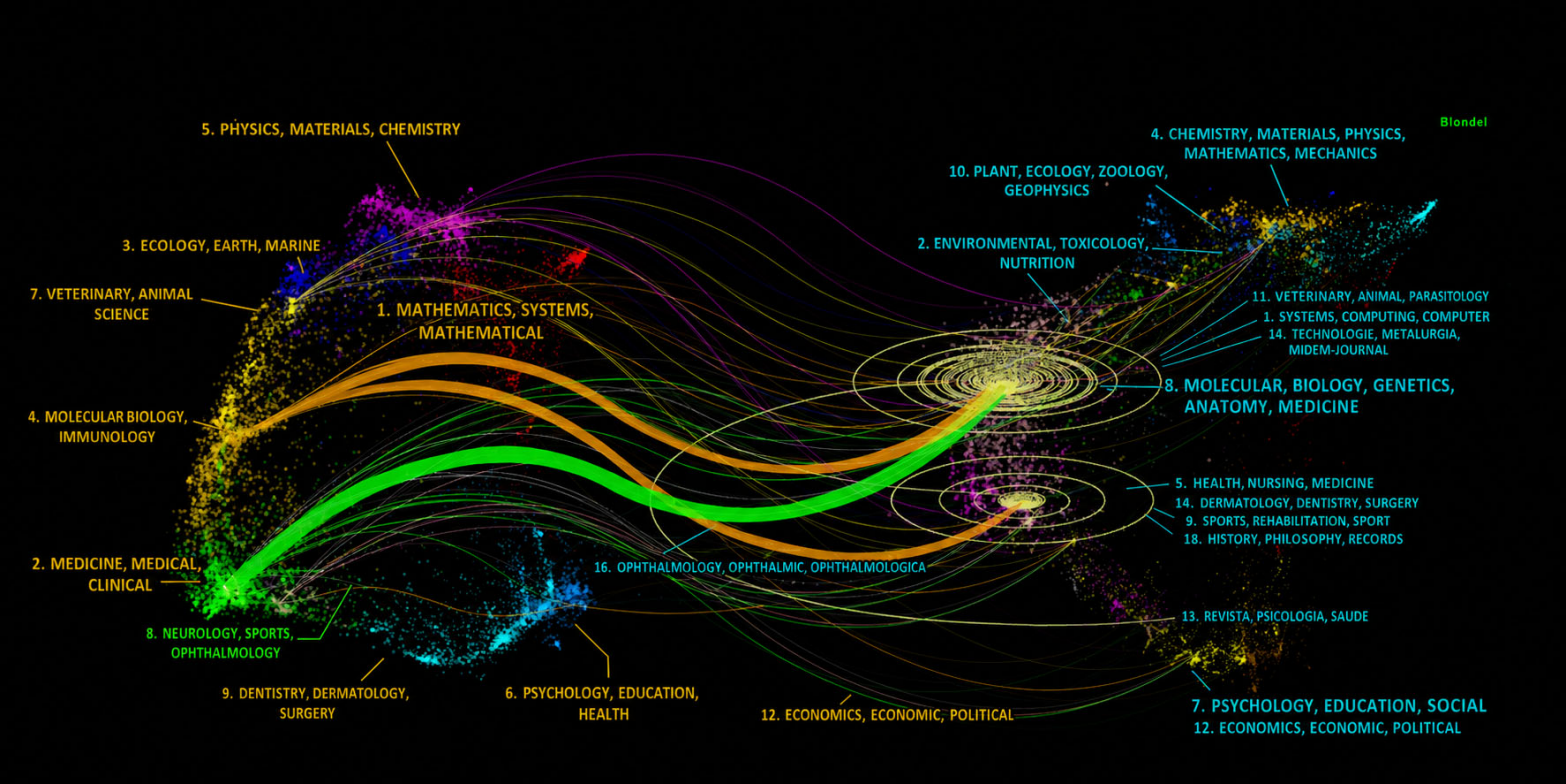
Dual-map overlay of journals on allergic diseases and B cells. This visualization employs a dual-map framework to simultaneously analyze the disciplinary distribution of scholarly journals (left map) and their citation relationships (right map). Each node represents a journal cluster, positioned based on topic similarity derived from bibliographic coupling or co-citation networks.

The top 10 journals by publication volume are shown in **Supplementary Figure 1** and include the Journal of *Allergy and Clinical Immunology*, *Frontiers in Immunology*, and *The Journal of Immunology*. The dual-map overlay of journals shows the distribution of relationships between journals, with citing journals on the left and cited journals on the right (31). As shown in **Figure 3**, two dominant citation paths emerged orange and green, One signifies that studies published in “*Molecular*, *Biology*, *Immunology*” citing “*Molecular*, *Biology*, *Genetics*, “ and the other shows that studies published “*Medicine*, *Medical*, *Clinical*” citing “*Molecular*, *Biology*, *Genetics*.” **Supplementary Table 5** lists the 10 most influential articles on allergic diseases and B cells—canonical works that delineate major research directions in the field.

### 3.3 Countries and institutions

**Figure 4:**
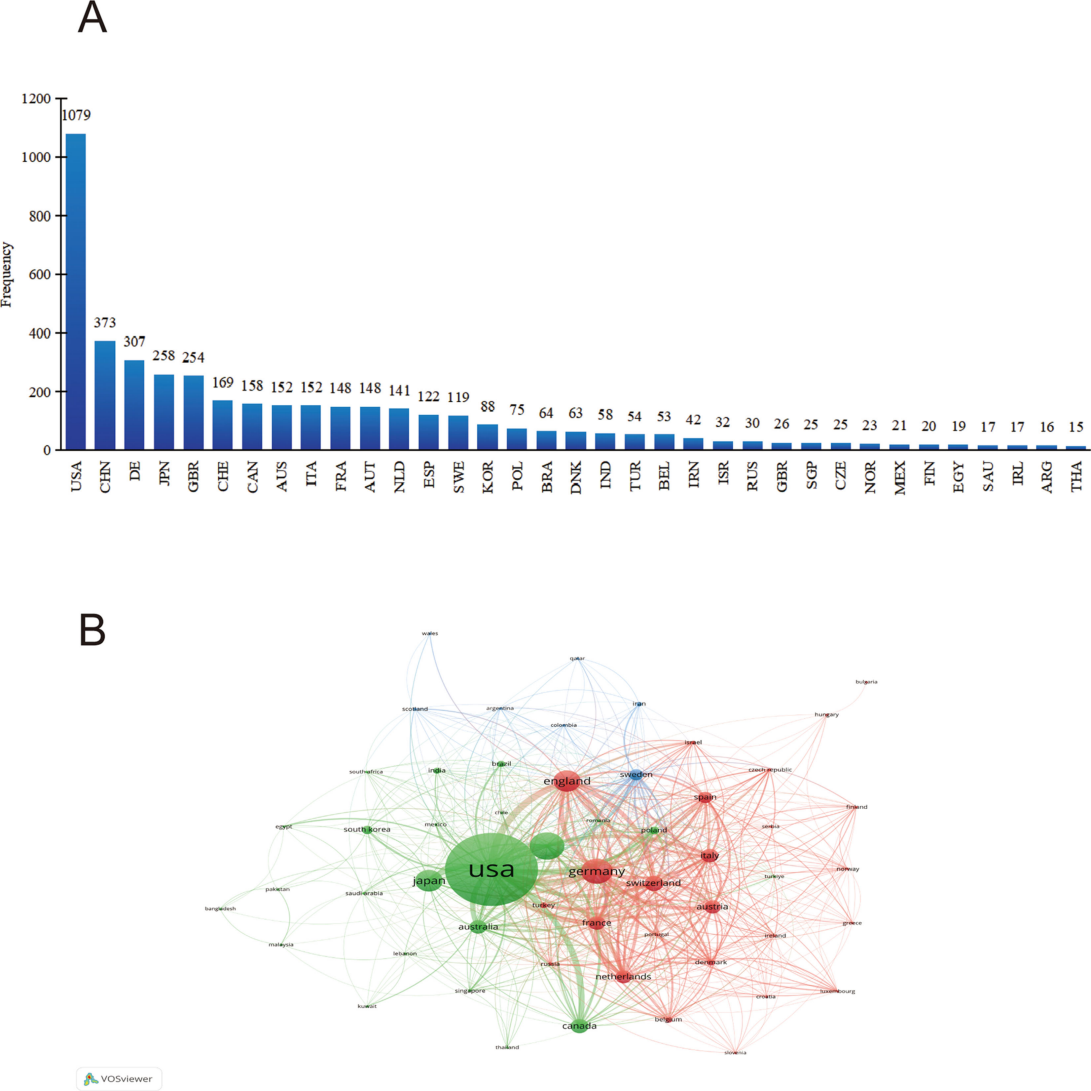
**(A)** Distribution of countries engaged in allergic diseases and B cells research. **(B)** Cooperation map of countries associated with research on allergic diseases and B cells.

Using VOSviewer and CiteSpace, we mapped international collaboration from 2005 to 2024. In total, 55 countries and at least 173 institutions produced 3, 084 publications. As shown in **Figure 4**, the United States was the most active country in international collaboration, followed by Germany, England, Switzerland, and France.

**Figure 5:**
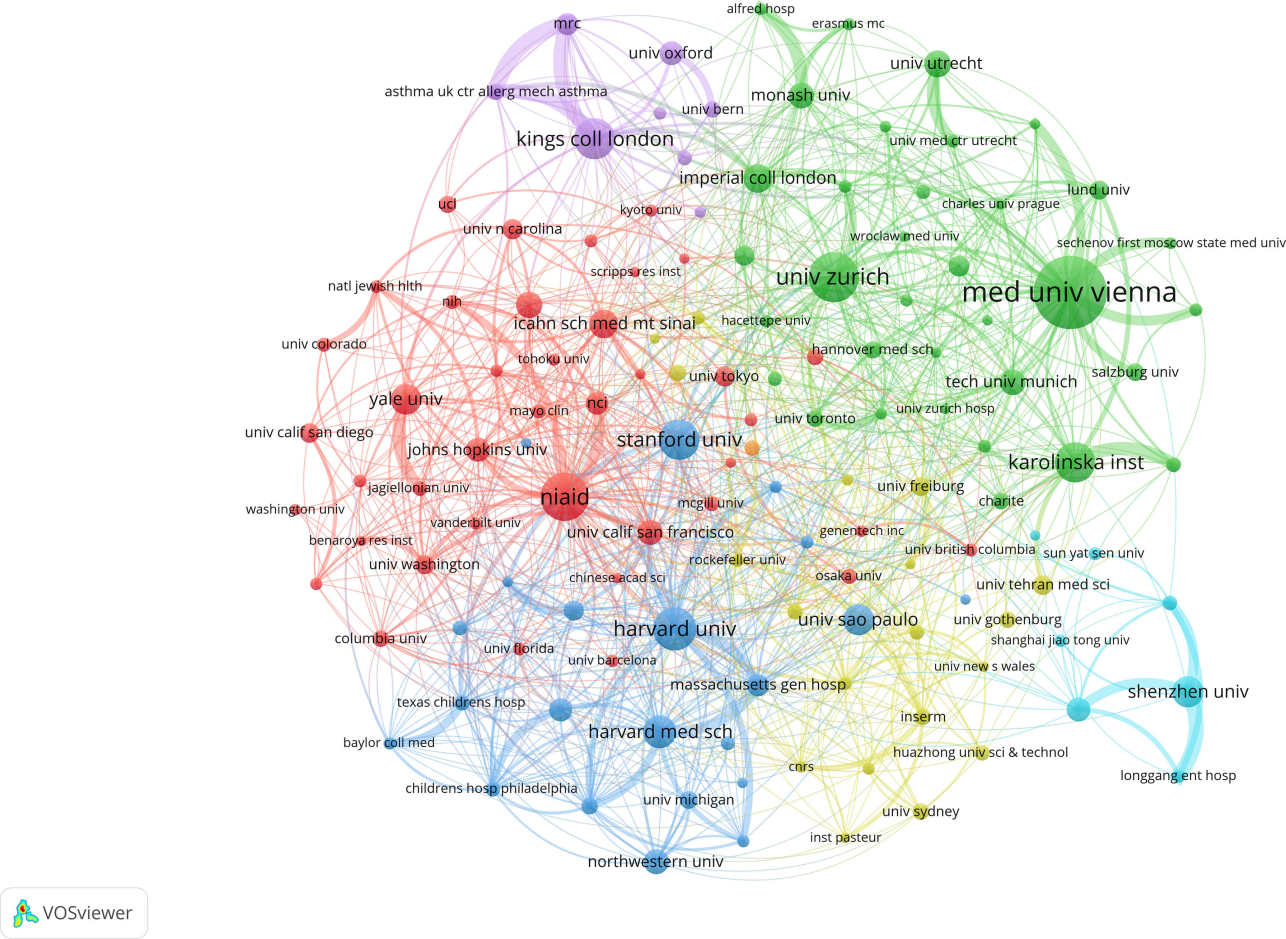
Institutions engaged in the research on allergic diseases and B cells.

As shown in **Figure 5**, the top five by publication volume were the Medical University of Vienna, University of Zurich, National Institute of Allergy and Infectious Diseases, Harvard University, and King’s College London. The top five by citation frequency were the University of Zurich, Harvard University, NIAID, Duke University, and King’s College London. All leading institutions were located in Europe or the United States, underscoring their central role in research on the interface of allergic diseases and B-cell biology. Dense collaboration links highlight sustained collective efforts to advance mechanistic understanding and therapeutic strategies. While Europe and the United States led overall output, contributions from other regions have grown, fostering the globalization of the field.

### 3.4 Authors

**Figure 6:**
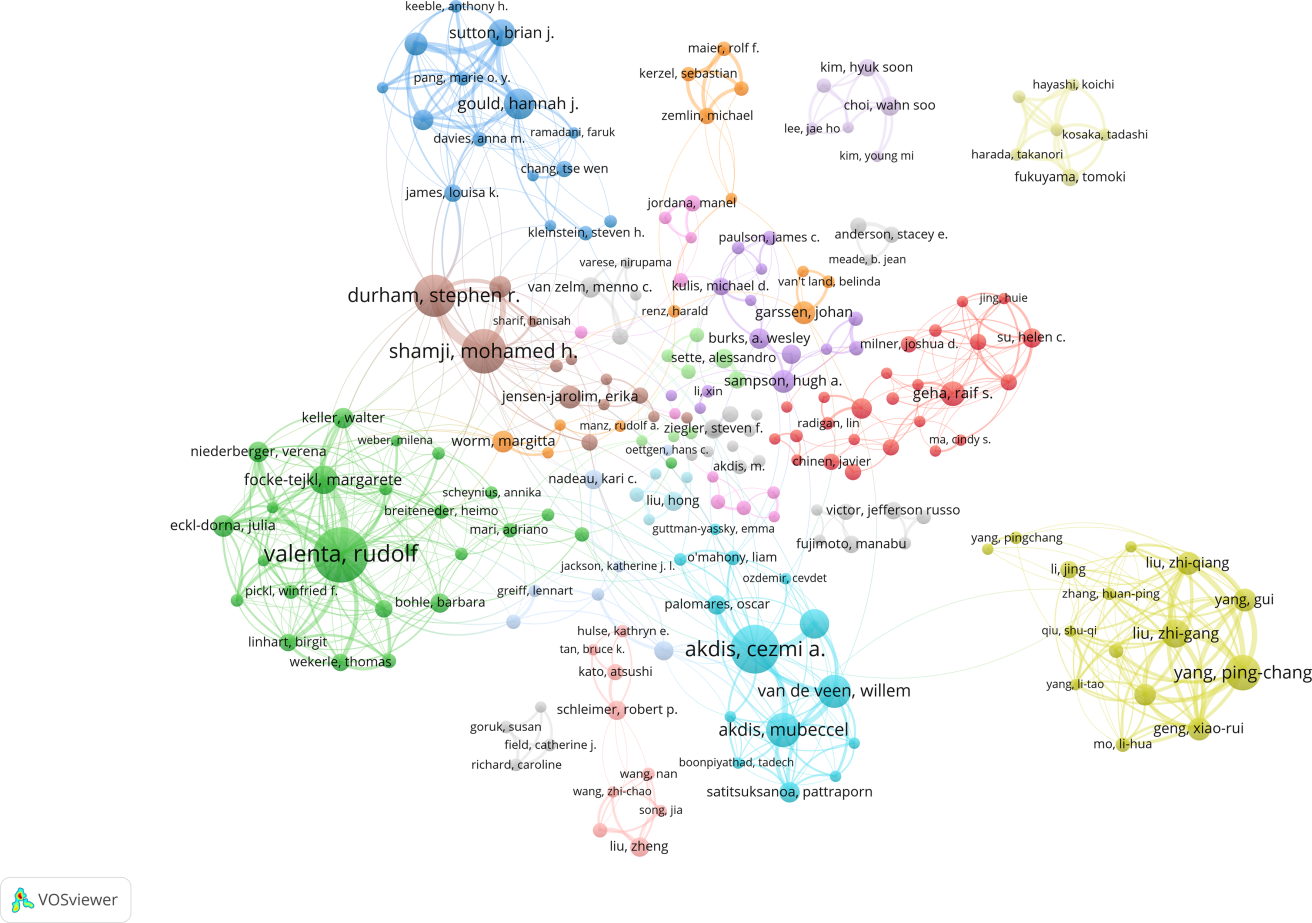
Joint mapping of the productive authors who published studies on allergic diseases and B cells from 2005 to 2024.

The top 10 authors contributed 257 papers related to allergic diseases and B cells; three authors each published >100 relevant articles (**Supplementary Table 6**). Valenta, Rudolf had the highest output (42 publications), followed by Akdis, Cezmi A. (35 publications) and Shamji, Mohamed H. (31 publications). Using VOSviewer (v1.6.18) and excluding authors with <5 co-occurrences, 232 authors met the threshold, yielding 213 nodes in 13 clusters. As shown in **Figure 6**, Node size reflects publication volume; edge thickness indicates collaboration strength. Among the top 10 collaborative subnetworks, seven authors were based in Europe, two in the United States, and one in China. Collaboration tended to be clustered—often reflecting work within the same research groups.

### 3.5 Keyword analysis

**Figure 7:**
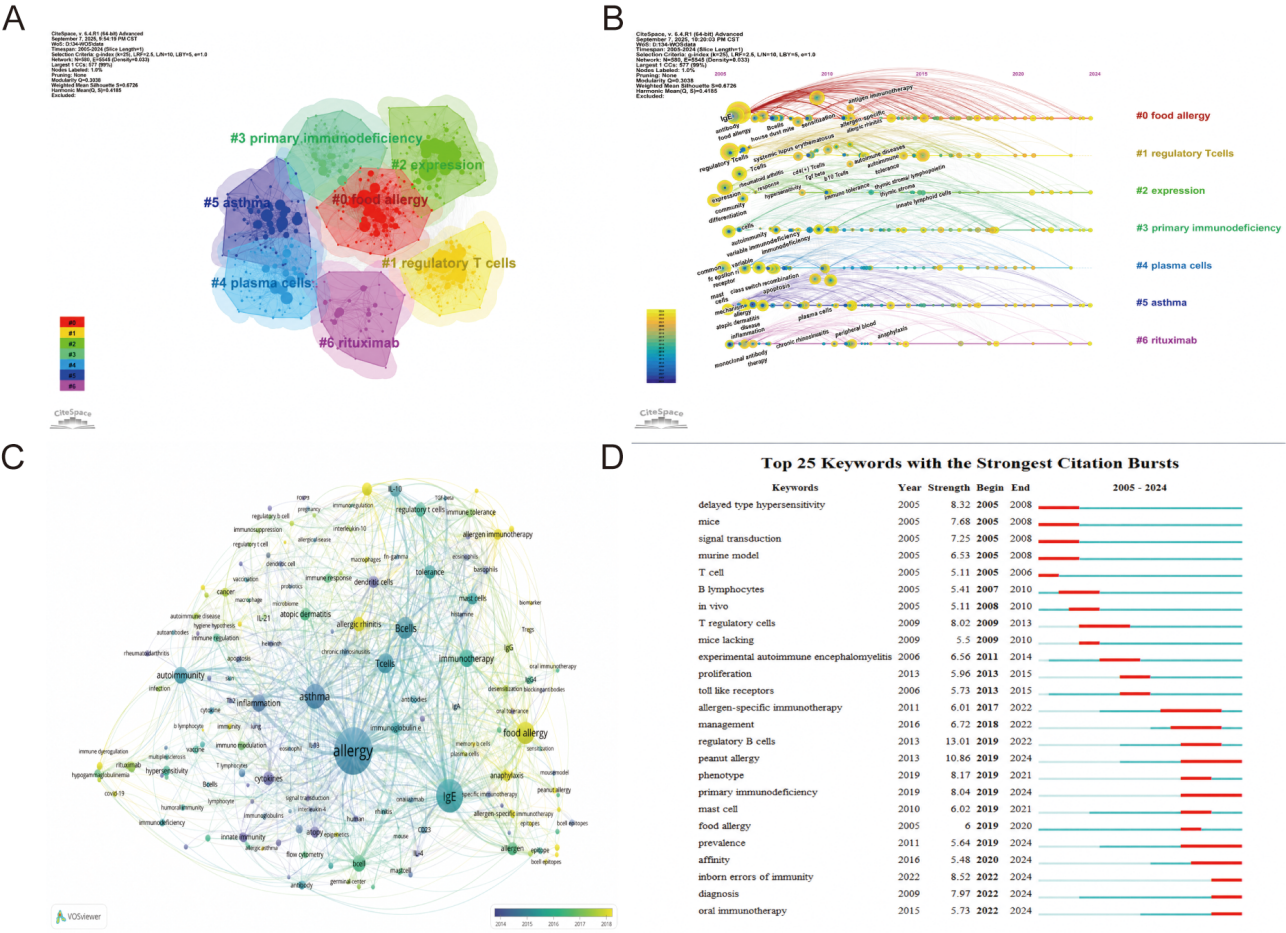
**(A)** Use CiteSpace’s keyword clustering to analyze research on allergic diseases and B cells. **(B)** Timeline analysis of keyword clusters in allergic diseases and B cell research in CiteSpace. **(C)** Keyword co-occurrence diagram of allergic diseases and B cells in VOSviewer. **(D)** CiteSpace visually presents the 25 keywords with the largest increase in citations in allergic diseases and B cell research from 2005 to 2024.

VOSviewer and CiteSpace were used to construct keyword co-occurrence networks. **Figure 7A** identified seven major clusters from 2005 to 2024: #0 ‘food allergy, ‘ #1 ‘regulatory T cells, ‘ #2 ‘expression, ‘ #3 ‘primary immunodeficiency, ‘ #4 ‘plasma cells, ‘ #5 ‘asthma, ‘ and #6 ‘rituximab.’ Taken together, these clusters suggest a conceptual progression from disease-centered themes (#0, #5) toward immune regulation (#1), molecular profiling (#2), immune-dysregulation contexts (#3), humoral effector and memory biology (#4), and exploratory B-cell-targeted therapy (#6). This pattern indicates that the field has broadened beyond descriptive disease associations to include mechanistic, molecular, and translational research themes, while the precise biological roles of B cells in allergy remain an active area of investigation.

The timeline overlay (**Figure 7B**) showed that several themes, including ‘food allergy, ‘ ‘regulatory T cells, ‘ ‘expression, ‘ ‘plasma cells, ‘ and ‘asthma, ‘ persisted across much of the study period, whereas ‘innate lymphoid cells’ emerged more prominently in later years, suggesting a shift toward broader cellular-network and tissue-context perspectives.

**Figure 7C** extracts a total of 173 keywords; six appeared >100 times and 22 appeared >50 times. The top 25 co-occurring keywords are listed in **Supplementary Table 7** and include b cells (n=643), t cells (n=443), expression (n=391), dendritic cells (n=309), responses (n=265), regulatory t cells (n=257), food allergy (n=241), activation (n=239), asthma (n=190), mast cells (n=188), and b cell (n=181). Food allergy emerged as a core theme linking allergic disease research and B-cell biology.

Keyword burst detection (**Figure 7D**) identified terms with rapid increases in attention over defined intervals. ‘Delayed type hypersensitivity’ was prominent in the earlier period, whereas ‘regulatory B cells’ showed a strong later burst, consistent with growing research interest in immune regulation and tolerance.

### 3.6 Analysis of clinical trial progress in PubMed database

**Figure 8:**
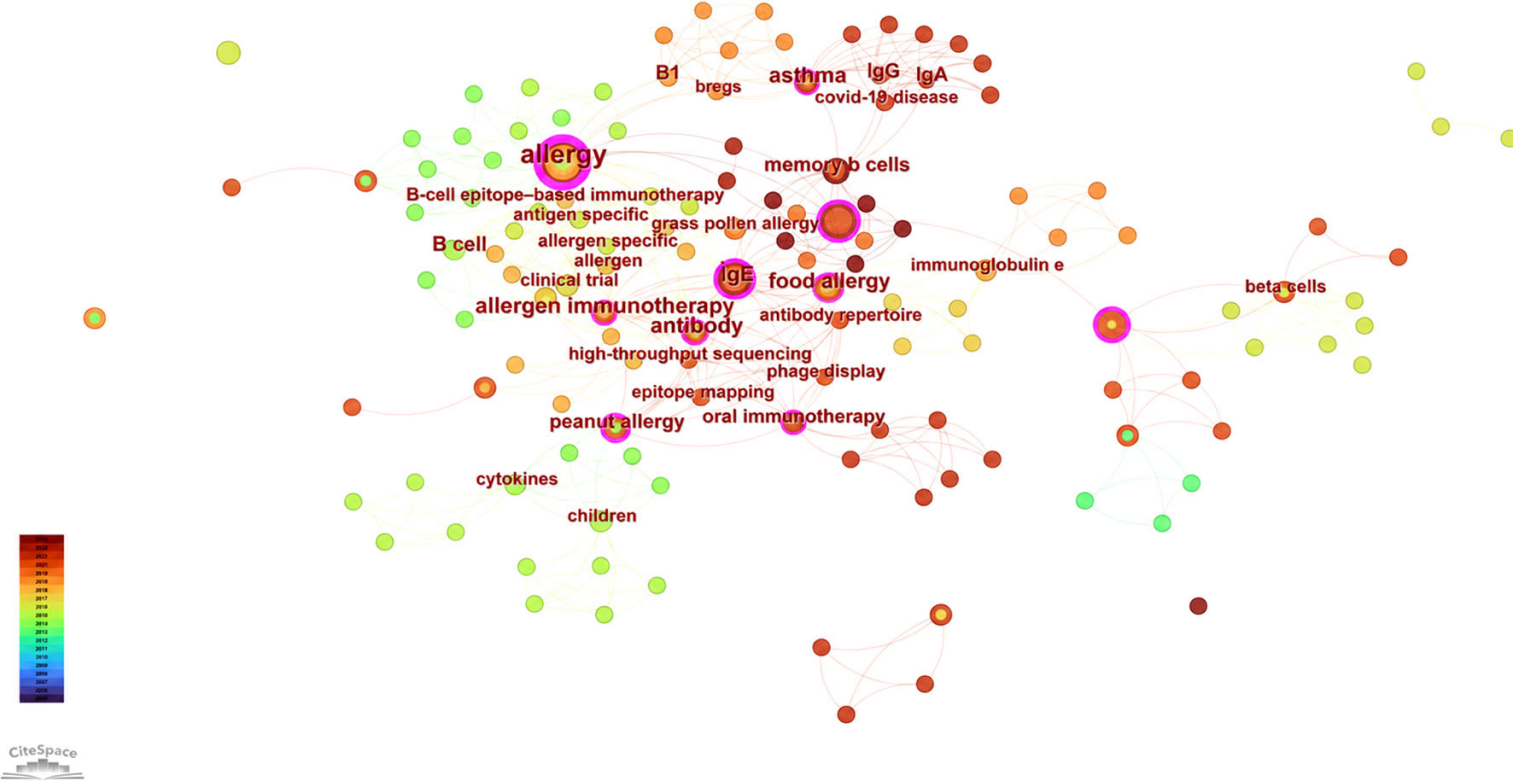
Co-occurrence graph of keywords in the PubMed database on clinical trial progress in allergic diseases and B cell research.

**Figure 9:**
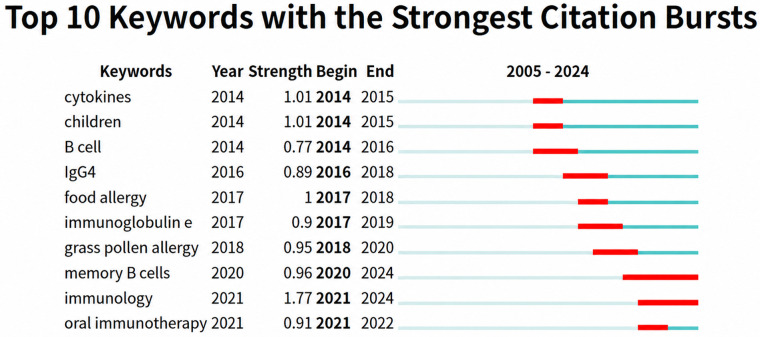
A visual map of the 10 keywords cited in the PubMed database with the strongest progress in clinical trials from 2005 to 2024.

PubMed is recognized as a high-quality medical database that includes many well-conducted clinical trials (32). Therefore, we used PubMed to analyze the clinical progression of B cells in allergic diseases to provide clinical researchers in this field with insights into current research hotspots and emerging trends. Keywords are an important window to understand the focus and frontiers of research fields. By constructing keyword burst detection (**Figure 8**) and keyword co-occurrence graphs (**Figure 9**), this study drew several conclusions on the progress of clinical research in allergic diseases and B cells.

#### 3.6.1 Allergic disease and IgE from memory B cells

Keyword bursts for “immunoglobulin e, “ “immunology, “ and “memory B cells” support the notion—based on PubMed clinical-trial records—that inhaled allergens can induce formation of lung-resident memory B cells (MBCs). In particular, IgG1^+^ MBCs may undergo IgE class-switch recombination under IL-4 produced by lung-resident Th2 cells. Upon re-exposure to allergen, these lymphocytes can generate IgE locally within the airways.

#### 3.6.2 B-cell subsets in allergic asthma

Beyond the three terms above, the co-occurrence map highlighted tight links among “asthma, “ “b cell, “ and “antibody.” Experimental evidence indicates that cell-derived IL-10 promotes epithelial and dendritic-cell responses to the asthma allergen house dust mite (HDM) (33). Bcl-3 functions as a negative regulator of B-cell-derived IL-10; by limiting IL-10 production during the sensitization phase, Bcl-3 constrains HDM-induced asthma. These data suggest that targeting IL-10 and Bcl-3 signaling to prevent allergen sensitization could be a promising therapeutic strategy (34).

#### 3.6.3 Design considerations and future directions

Clinical-trial design in this domain still faces notable limitations: most studies are single-center with small sample sizes. To meet international standards of evidence-based medicine, future trials should adopt contemporary methodological frameworks—larger sample sizes, multi-center designs, and objective endpoints (35). For example, standardized measurement of B-cell subsets and counts in peripheral blood (36), quantification of IgM, IgG (37), and cytokines such as IL-10 and IL-35 (38), implemented across multiple sites, would strengthen inference. Consequently, supplementing randomized controlled trials with large-scale, multi-center studies anchored by objective outcome measures is likely to become an important trend (39).

## 4 Discussion

This 20-year bibliometric overview suggests that research on allergic diseases and B cells has evolved from a relatively narrow focus on immunoglobulin E (IgE) and clinical phenotypes toward broader interest in B-cell subsets, tissue context, and translational applications. The sustained growth in publications and citations indicates an expanding research community and continuing attention to this topic. The dual-map overlay further suggests close exchange between basic immunology journals and clinical allergy journals, highlighting the translational orientation of the field. The concentration of the most prolific and most cited institutions in Europe and the United States is consistent with long-standing research consortia in allergy and immunology and with the presence of leading groups that have driven fundamental advances in allergen immunotherapy (AIT), IgE memory, and regulatory B-cell biology.

### 4.1 B cells as the tissue “engine” of allergic memory

Two features of the present analysis are particularly noteworthy: the prominence of “food allergy, “ “asthma, “ and “plasma cells” within the keyword structure, and the sustained citation burst of “regulatory B cells” since 2015. Taken together, these findings suggest that the field has increasingly moved beyond a predominantly T cell-centered description of allergy toward greater interest in the contributions of B-cell lineages, tissue niches, and immunoregulatory processes (40).

First, external experimental studies provide a useful context for interpreting why tissue-associated B-cell biology has attracted growing attention (41). Prior work in allergic asthma and rhinitis has reported evidence consistent with local class-switch recombination and local IgE production in airway mucosa (42, 43). These observations challenged the earlier assumption that IgE is generated only in systemic lymphoid compartments and then trafficked back to target tissues (44). Although bibliometric analysis cannot confirm this model, the recurrent appearance of terms such as “plasma cells, “ “expression, “ and airway-related disease phenotypes in our results is consistent with sustained interest in tissue-localized antibody response. This alignment suggests that the field increasingly considers allergic inflammation within local microenvironments rather than solely through circulating biomarkers (45).

Second, A related line of investigation has focused on the cellular basis of persistent IgE memory. Studies using lineage-tracing, repertoire analysis, and functional modeling have proposed that non-IgE memory B-cell populations, particularly IgG1-associated memory cells, can contribute to the regeneration of IgE responses after allergen re-exposure under type 2 cytokine conditions. This framework has been used to reconcile the apparent rarity of bona fide IgE-positive memory B cells with the long-term persistence of IgE-mediated disease. In our bibliometric analysis, the emergence of terms related to memory B cells is compatible with this shift in emphasis (46, 47).

A corollary is that therapeutic interception at the level of tissue-resident memory B cells could durably depress IgE regeneration (48). Indeed, co-occurrence maps linking “asthma, “ “B cell, “ and “antibody” align with preclinical data in house dust mite models: B-cell-derived IL-10 modulates epithelial and dendritic cell responses during sensitization (49), whereas the transcriptional regulator Bcl-3 restrains IL-10, shaping downstream allergic inflammation (50). Although clinical transformation is still in its infancy, these data provide testable hypotheses for pre-sensitization prevention, such as enhancing B cell IL-10 or antagonizing Bcl-3 during seasonal sensitization periods (51, 52).

Overall, the persistence of clusters related to “plasma cells” and “expression” supports the interpretation that the field continues to invest substantial effort in understanding local antibody production, tissue-resident immune memory, and the regulation of allergic inflammation. In this context, the literature increasingly frames tissue-localized class switching, transient plasma-cell responses, and memory B-cell reactivation as interconnected processes relevant to disease recurrence (53), This perspective also offers one possible explanation for why therapies that neutralize circulating IgE can produce rapid clinical benefits yet may require continued administration in some settings (54).

### 4.2 Regulatory B cells and the architecture of tolerance

The strongest burst term since 2015 in our results, “regulatory B cells, “ is consistent with the broader literature showing that human IL-10–producing B cells, are integral to clinical tolerance (55). In allergen immunotherapy, these cells suppress antigen-specific T-cell responses and preferentially undergo class switching to IgG4 (56), yielding “blocking” antibodies that compete with IgE for allergen and attenuate FcϵRI-mediated activation (57). This dual functionality—active suppression and Fc competition—explains why increases in bioactive allergen-specific IgG correlate better with durable clinical benefit.

**Figure 10:**
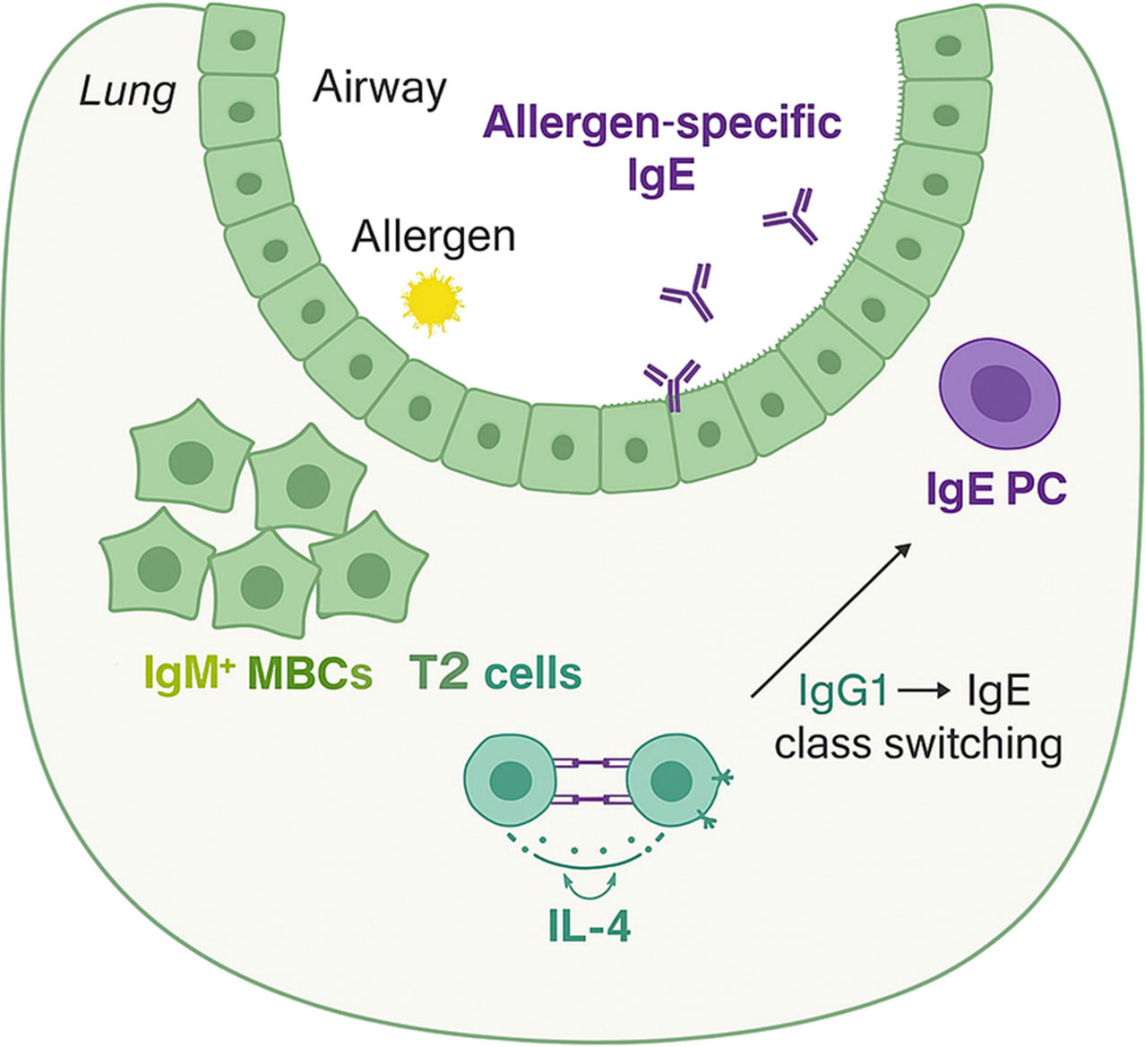
Mechanism of pulmonary memory B cells maintaining respiratory allergic reactions. (created with Biorender.com).

Recent work adds further nuance: although IgG4 has been the canonical “tolerance antibody, “ longitudinal AIT cohorts show dynamic roles for IgG1 and IgG4, with early suppression often dominated by IgG1 activity and later maintenance by IgG4, paralleling the maturation of affinity and epitope breadth (58). A long-standing puzzle in allergy immunology has been how durable IgE responses are maintained, given that IgE^+^ memory B cells are either extremely rare or absent (59). As shown in **Figure 10**, the critical insight from Nelson et al. is that IgG1^+^ memory B cells function as the precursors for IgE-secreting plasma cells in the lung. They showed that, in their model, IgG1^+^ memory B cells in lung tissue switched to IgE when exposed to IL-4 from lung Th2 cells in ex vivo co-culture (60). Our keyword burst analysis is consistent with recent research results, with “regulatory B cells” shifting from static isotype titers to functional readings in the AIT mechanism “ (61), “ IgE promotes allergen binding IgE-FAB” (62), “Basophil activation test” (63), “Promoted antigen presentation test to quantify CD23-dependent complexes” (64), etc. have become research hotspots.

Mechanistically, the low-affinity IgE receptor CD23 on B cells is a central conduit for these processes (65). CD23 governs uptake of IgE–allergen immune complexes and facilitates antigen presentation (66), but at the same time provides a non-inflammatory route that can be co-opted by “blocking” IgG to sequester complexes away from FcϵRI pathways (67). Structural and cellular studies show that CD23 density on B cells scales with serum IgE levels and determines the efficiency of IgE-facilitated uptake; crystallographic work further reveals allosteric coupling between the CD23–IgE interface and FcϵRI binding (68). Together, these insights sharpen understanding of how AIT-induced IgG can blunt CD23-mediated T-cell activation and why CD23 metrics are attractive biomarkers in trials.

Finally, our overlay analysis highlighting “innate lymphoid cells” as an emergent term is not a distraction from B-cell immunoregulation; rather, it represents a missing piece. During grass-pollen AIT, a population of KLRG1 type 2 innate lymphoid cells (ILC2s), acquires IL-10 competence in response to IL-33 and retinoic acid and is associated with clinical response (69). These IL-10^+^ ILC2s attenuate Th2 responses and maintain epithelial integrity, forming a regulatory circuit with Breg and Treg compartments (70). I Therefore, the simultaneous prominence of terms related to regulatory B cells and innate lymphoid cells may indicate growing interest in multicellular models of tolerance rather than in isolated cell populations. This broader framing may be especially useful for understanding why successful immunotherapy is increasingly discussed in terms of coordinated regulatory circuits. From maps to medicine: diagnostics and therapeutics shaped by B-cell biology.

### 4.3 From maps to medicine: diagnostics and therapeutics shaped by B-cell biology

#### 4.3.1 Diagnostic translation

The centrality of B cells naturally foregrounds antibody specificity and function. Component-resolved diagnostics (CRD) leverage molecular allergens to delineate genuine sensitization from cross-reactivity and to estimate risk (71). However, both our comprehensive analysis and the EAACI position paper emphasize that CRD alone is not sufficient as a predictive biomarker of AIT efficacy (72). In contrast, functional indicators of B cell-mediated competition, such as IgE-FAB inhibition and basophil activation, can be captured, which is more relevant to clinical outcomes and can be standardized for use in multi-center trials (73). The persistence of keywords related to “expression” and “activation” in our analysis is consistent with this transition from static serological measurements toward functional immunological assessment.

#### 4.3.2 Immunotherapy continuum

On the therapeutic side, AIT remains the principal disease-modifying intervention for respiratory allergy and has therefore occupied a central place in the literature captured by our analysis. Its reported mechanisms include early effector-cell desensitization (74), progressive expansion of regulatory pathways, induction of IL-10-associated B-cell responses, and the accumulation of allergen-specific IgG antibodies with blocking activity (75). Contemporary studies also emphasize the durability of benefit after treatment cessation in some patients, which has directed attention toward the reprogramming of immunological memory and tissue microenvironments (76).

#### 4.3.3 Development based on B cell epitopes

This conceptual shift is also reflected in the development of B-cell epitope-based vaccine approaches designed to induce protective IgG responses while minimizing the risk of boosting IgE (77). Recombinant constructs such as BM32 have been discussed as examples of how nonallergenic B-cell epitope design may support favorable efficacy and safety profiles in grass-pollen allergy (78). From a bibliometric perspective, such approaches illustrate how intensified attention to B-cell biology can correspond to innovation in vaccine design.

#### 4.3.4 Anti-IgE biologics in food allergy

The clinical-trial frontier profiled in our analysis shows a decisive turn toward B-cell–informed interventions in food allergy. In 2024, a phase III trial demonstrated that omalizumab monotherapy increased reaction thresholds across multiple foods in patients as young as 1 year and outperformed multi-allergen oral immunotherapy (OIT) in a subsequent head-to-head stage, largely because of OIT-associated adverse events. Mechanistically, neutralizing free IgE reduces FcϵRI occupancy on effector cells and, by altering feedback on CD23^+^ B cells, can dampen facilitated antigen presentation, again underscoring B-cell circuitry as a key component of the drug’s benefit. The FDA approval that followed is therefore not merely a pharmacological milestone; it corroborates our bibliometric observation that clinically relevant progress in allergy increasingly depends on manipulating B-cell and IgE axes.

The clinical-trial landscape represented in our analysis also appears to reflect increasing interest in B-cell- and IgE-oriented interventions for food allergy (79). Reports of phase III studies of omalizumab monotherapy in multi-food allergy, as well as comparative analyses involving oral immunotherapy, have heightened attention to strategies that reduce free IgE and alter downstream effector-cell activation (80). These advances are highly relevant to the broader interpretation of our bibliometric findings because they demonstrate how the IgE axis continues to shape translational priorities. At the same time, the significance of omalizumab should be understood through clinical-trial evidence and regulatory evaluation, whereas our bibliometric analysis simply shows that such B-cell/IgE-centered interventions have become more prominent within the literature.

### 4.4 Disease entities through the B-cell lens

#### 4.4.1 Asthma and airway disease

The sustained presence of “asthma, “ “plasma cells, “ and “innate lymphoid cells” among co-occurring terms reflects two converging narratives. First, airway tissues can generate IgE locally, and IgG1^+^ memory B-cell pools can reseed IgE upon allergen re-encounter, explaining chronicity and seasonal relapses (81). Second, IL-10 B cells and IL-10 ILC2 cells in the regulatory circuit can actively inhibit this cycle, especially under AIT (82). Its clinical significance is that endpoint indicators in asthma clinical trials should include proximal tissue indicators and functional serological indicators, such as mRNA expression levels in the nasal cavity or bronchus, memory B cell phenotypic analysis, and expression levels of IgE and IgD, not just symptom scores or serum IgE levels (83).

#### 4.4.2 Rhinitis phenotypes and local IgE

The keyword cluster including “food allergy, “ “asthma, “ and “primary immunodeficiency” also intersects with “local IgE” phenotypes. Local allergic rhinitis (LAR), a condition with negative systemic tests but positive nasal provocation and mucosal IgE, has been substantiated by prospective follow-up studies showing that it is a consistent, independent phenotype, not merely a prodrome of systemic atopy (44). Similarly, chronic rhinosinusitis with nasal polyps often exhibits local IgE and related B-cell activation in polyp tissue (84). These entities reinforce the need for site-specific sampling and for recognizing that B-cell class switching and effector functions can be “entopic” even in the absence of systemic sensitization.

#### 4.4.3 A field coalescing on B-cell–centric precision medicine

Taken together, our findings depict a field that is moving toward more precise, B-cell-informed models of allergic disease while also expanding its translational ambitions. The rise of “regulatory B cells, “ the continuity of “food allergy, asthma, plasma cells, “ and the recent hotspot “innate lymphoid cells” chart a path in which tissue-localized B-cell memory, IgE regeneration, and regulatory circuits explain both disease persistence and successful therapy. Diagnostics are evolving from extract-based sensitization screens toward component-resolved and functional assays that reflect B-cell competition with IgE. Treatment methods are tending to be two complementary strategies: first, memory B cells can change the structure of IgE effector functional areas and reduce the affinity of IgE with FC and CD23, thereby reducing the body’s sensitization and inflammatory response (85); second, regulatory B cells can reduce airway hyperresponsiveness and lung remodeling in allergic diseases (86). These two paths are not developing in parallel; they are step-by-step steps towards precision allergy medicine based on B-cell immunology.

## 5 Conclusions

This 20-year bibliometric analysis of 3, 084 WoSCC indexed articles and 71 PubMed-registered clinical trials shows that research on allergic diseases and B cells has expanded steadily in volume, influence, and conceptual sophistication. The field has shifted from a predominantly IgE- and phenotype-focused view toward a tissue- and systems-level understanding of B-cell biology, with intensive cross-talk between basic immunology and clinical allergy journals. Leading institutions in Europe and the United States dominate output and collaboration, although contributions from other regions are increasing.

Keyword clustering and burst analyses identify food allergy, asthma, and plasma cells as stable thematic cores, while regulatory B cells and innate lymphoid cells have emerged as major hotspots. These patterns support a B-cell–centric paradigm in which tissue-resident memory B cells and local class-switch recombination sustain IgE production in affected organs, whereas IL−10–producing regulatory B cells contribute critically to immune tolerance and the efficacy of allergen immunotherapy. The prominence of terms such as “expression, “ “activation, “ and “memory B cells” reflects a shift toward functional and tissue-resolved assessments of B-cell activity.

Analysis of clinical trials indicates that translation of B-cell biology into therapeutic innovation, through anti-IgE biologics, optimized allergen immunotherapy, and early B-cell–targeted strategies is underway but limited by small, often single-center study and heterogeneous endpoints. Future work should prioritize multicenter study, adequately powered trials incorporating standardized B-cell phenotyping, longitudinal monitoring of allergen-specific IgE and IgG, and harmonized clinical outcomes.

## Data Availability

The original contributions presented in the study are included in the article/**Supplementary Material**. Further inquiries can be directed to the corresponding author.
