# Supplementary material for: Studies on allergic diseases and B cells in the past 20 years: a bibliometric analysis via CiteSpace and VOSviewer
Source: Front Immunol. 2026 May 14;17:1760388. doi: 10.3389/fimmu.2026.1760388 (PMC13215865; doi:10.3389/fimmu.2026.1760388)

***Supplementary Material***

**Supplementary Tables**

**Table S1. Summary of Data Sources and Selection Criteria from the Web of Science Core Collection Database**

| **Category** |  | **Specific Standard requirement** |
| --- | --- | --- |
| Research database |  | Web of Science core collection |
| Citation indexes |  | Science Citation Index Expanded |
| Searching period |  | 2005-01-01 to 2024-12-31 |
| Developed by |  | Weiyuan Mai, Xiaoqu Chen, Wanlin Ye |
| Language |  | “English” |
| Keywords | Keywords for searching | TS = (allergy OR "allergic disease" OR "allergic diseases" OR atopy OR "atopic disease" OR "atopic diseases" OR anaphylaxis OR "anaphylactic response" OR "anaphylactic responses" OR "anaphylactic reaction" OR "anaphylactic reactions" OR hypersensitivity OR "hypersensitive response" OR "hypersensitive responses" OR "hypersensitive reaction" OR "hypersensitive reactions") AND TS = ("b cell" OR "b cells" OR "b-lineage cell" OR "b-lineage" OR "b lymphocytes" OR "beta cells") and English (Languages) and Article or Review Article (Document Types) |
| Data extraction |  | Export with full records and cited references in plain  text format |
| Sample size |  | 3084 |

**Table S2. Summary of Data Sources and Selection Criteria from the PubMed Databas**

| **Category** | **Specific Standard requirement** |
| --- | --- |
| Research  database | PubMed |
| Citation  indexes | Science Citation Index Expanded |
| Searching  period | 2005-01-01 to 2024-12-31 |
| Developed  by | Weiyuan Mai, Xiaoqu Chen, Wanlin Ye |
| Language | “English” |
| Keywords | ((((((((((((((((allergy[Title/Abstract]) OR ("allergic disease"[Title/Abstract])) OR ("allergic diseases"[Title/Abstract])) OR (atopy[Title/Abstract])) OR ("atopic disease"[Title/Abstract])) OR ("atopic diseases"[Title/Abstract])) OR (anaphylaxis[Title/Abstract])) OR ("anaphylactic response"[Title/Abstract])) OR ("anaphylactic responses"[Title/Abstract])) OR ("anaphylactic reaction"[Title/Abstract])) OR ("anaphylactic reactions"[Title/Abstract])) OR (hypersensitivity[Title/Abstract])) OR ("hypersensitive response"[Title/Abstract])) OR ("hypersensitive responses"[Title/Abstract])) OR ("hypersensitive reaction"[Title/Abstract])) OR ("hypersensitive reactions"[Title/Abstract])) AND (((((("b cell"[Title/Abstract]) OR ("b cells"[Title/Abstract])) OR ("b-lineage cell"[Title/Abstract])) OR ("b-lineage"[Title/Abstract])) OR ("b lymphocytes"[Title/Abstract])) OR ("beta cells"[Title/Abstract])) AND English[lang] AND ("2005/01/01"[PDAT] : "2024/12/31"[PDAT]) |
| Sample size | 71 |

**Table S3. Controlled vocabulary:**

| **allergy** | **b cell** |
| --- | --- |
| **allergic disease** | **b cells** |
| **allergic diseases** | **b-lineage cell** |
| **atopy** | **b-lineage** |
| **atopic disease** | **b lymphocytes** |
| **atopic diseases** | **beta cells** |
| **anaphylaxis** |  |
| **anaphylactic response** |  |
| **anaphylactic responses** |  |
| **anaphylactic reaction** |  |
| **anaphylactic reactions** |  |
| **hypersensitivity** |  |
| **hypersensitive response** |  |
| **hypersensitive responses** |  |
| **hypersensitive reaction** |  |
| **hypersensitive reactions** |  |

**The above controlled vocabulary comes from Pubmed's Mesh.**

**Table S4. Fleiss’ Kappa for Each Reviewer Compared with the Standard**

**Table S5. Top 10 most influential articles on allergic diseases and B cells.**


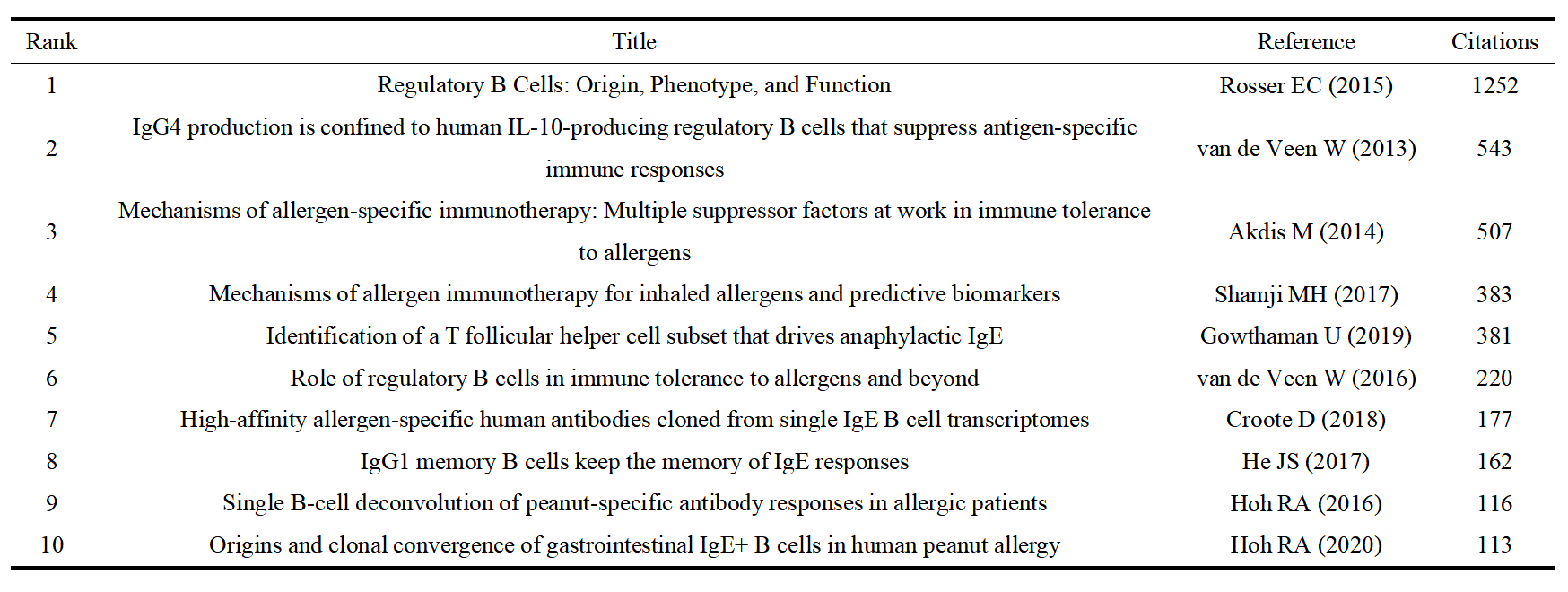


**Table S6. Top 10 authors on allergic diseases and B cells.**


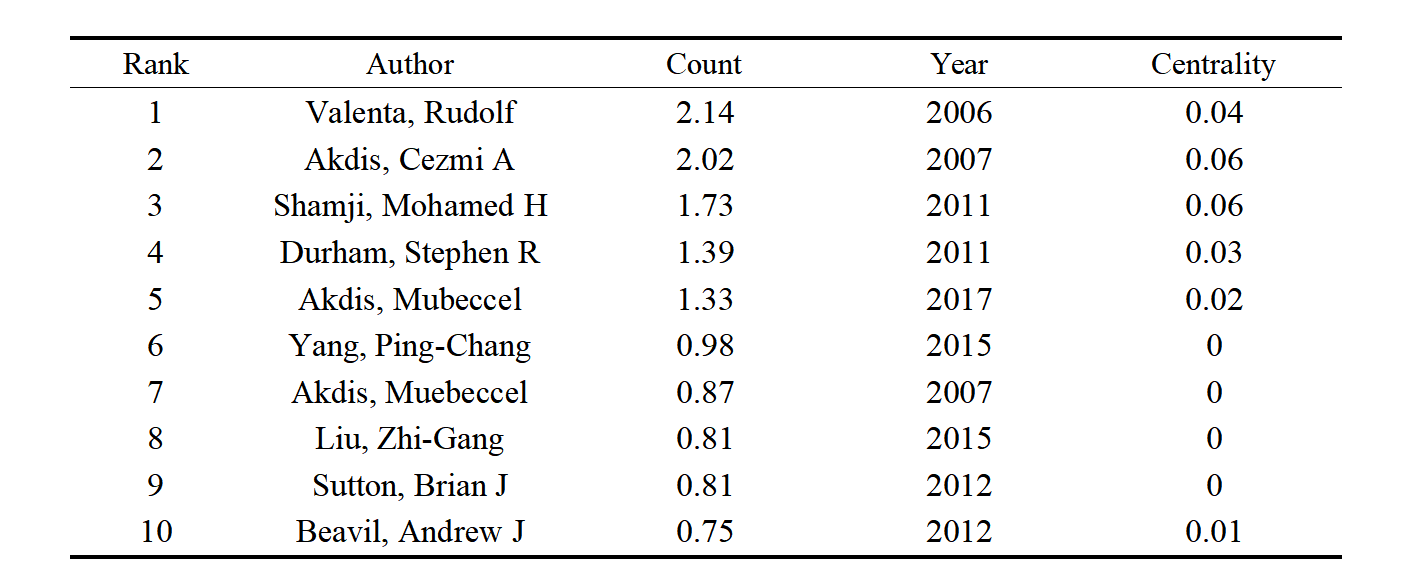


**Table S7. Top 25 keywords of allergic diseases and B cells.**


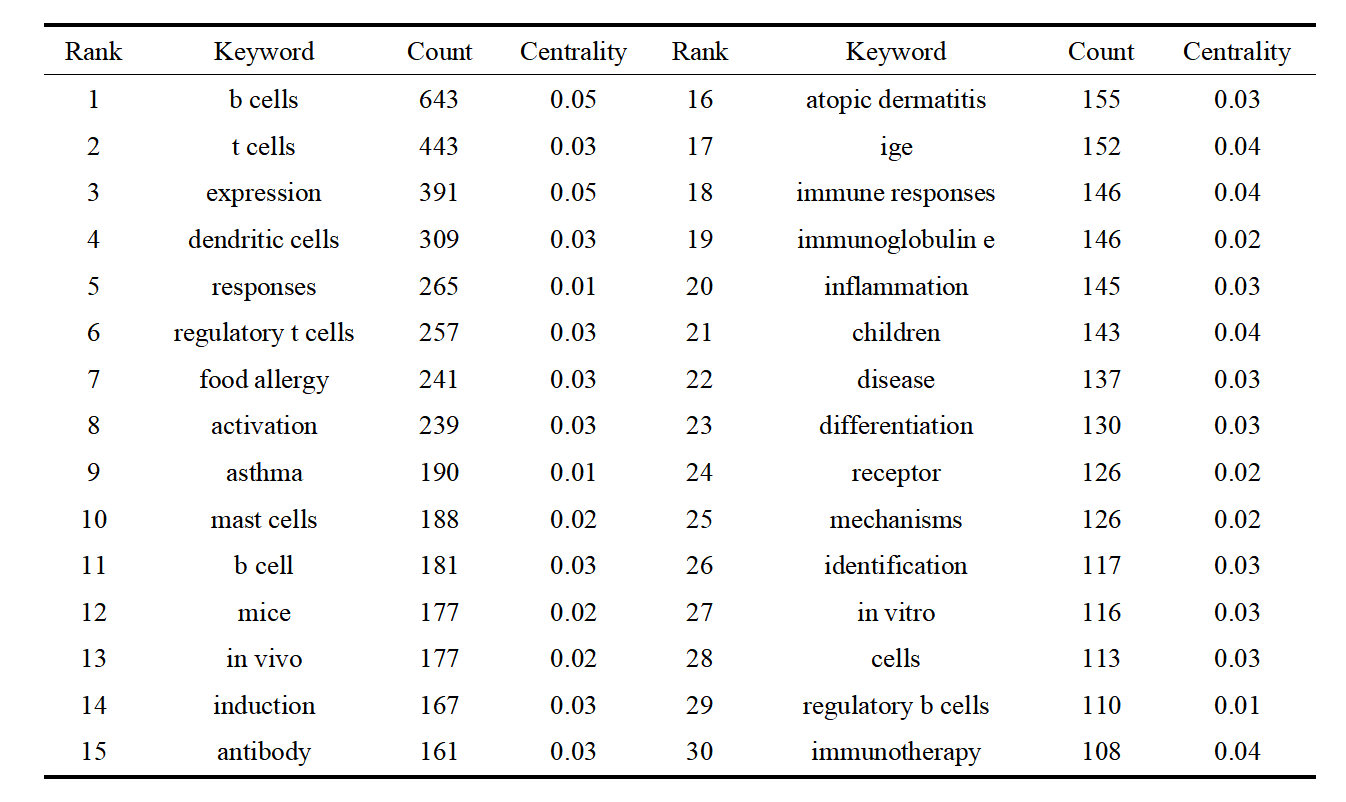


**Figure S1. Top ten prolific journals by number of articles published.**


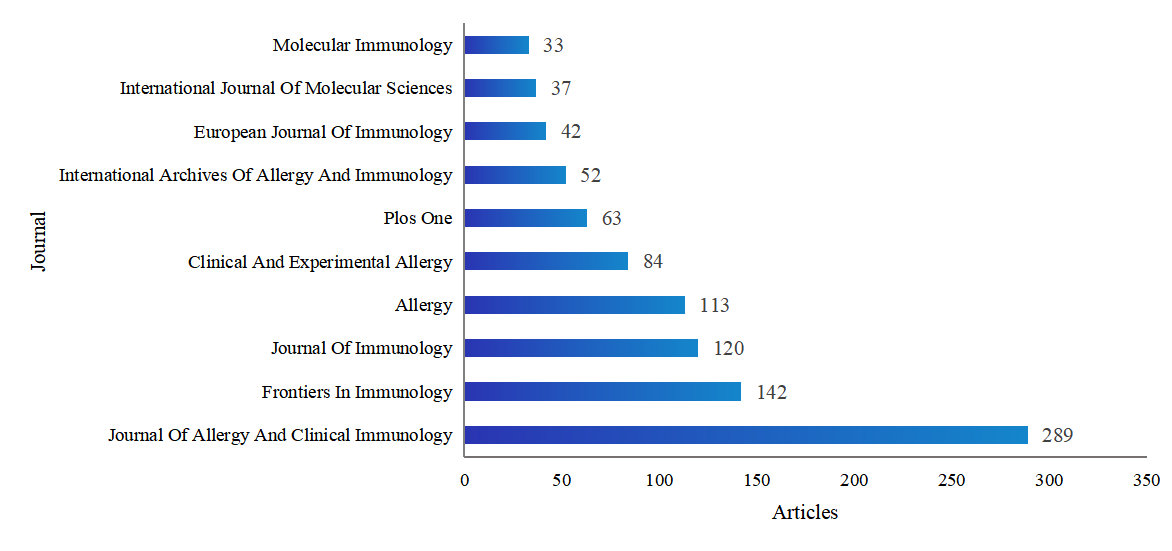

Supplement: Supplementary file 1 [file DataSheet1.docx]
